# Enhanced Vigilance Stability during Daytime in Insomnia Disorder

**DOI:** 10.3390/brainsci10110830

**Published:** 2020-11-07

**Authors:** Ariane Losert, Christian Sander, Michael Schredl, Ivonne Heilmann-Etzbach, Michael Deuschle, Ulrich Hegerl, Claudia Schilling

**Affiliations:** 1 Department of Psychiatry and Psychotherapy, Central Institute of Mental Health, Sleep laboratory, Medical Faculty Mannheim/Heidelberg University, 68159 Mannheim, Germany; ariane.losert@zi-mannheim.de (A.L.); michael.schredl@zi-mannheim.de (M.S.); Ivonne.Heilmann-Etzbach@zi-mannheim.de (I.H.-E.); michael.deuschle@zi-mannheim.de (M.D.); 2 Department of Psychiatry and Psychotherapy, University Leipzig Medical Center, 04103 Leipzig, Germany; Christian.Sander@medizin.uni-leipzig.de; 3 Department of Psychiatry, Psychosomatics, and Psychotherapy, Goethe-Universität Frankfurt am Main, 60528 Frankfurt am Main, Germany; Ulrich.Hegerl@kgu.de

**Keywords:** insomnia, arousal, vigilance, resting EEG, RDoC, polysomnography

## Abstract

Central nervous hyperarousal is as a key component of current pathophysiological concepts of chronic insomnia disorder. However, there are still open questions regarding its exact nature and the mechanisms linking hyperarousal to sleep disturbance. Here, we aimed at studying waking state hyperarousal in insomnia by the perspective of resting-state vigilance dynamics. The VIGALL (Vigilance Algorithm Leipzig) algorithm has been developed to investigate resting-state vigilance dynamics, and it revealed, for example, enhanced vigilance stability in depressive patients. We hypothesized that patients with insomnia also show a more stable vigilance regulation. Thirty-four unmedicated patients with chronic insomnia and 25 healthy controls participated in a twenty-minute resting-state electroencephalography (EEG) measurement following a night of polysomnography. Insomnia patients showed enhanced EEG vigilance stability as compared to controls. The pattern of vigilance hyperstability differed from that reported previously in depressive patients. Vigilance hyperstability was also present in insomnia patients showing only mildly reduced sleep efficiency. In this subgroup, vigilance hyperstability correlated with measures of disturbed sleep continuity and arousal. Our data indicate that insomnia disorder is characterized by hyperarousal at night as well as during daytime.

## 1. Introduction

Chronic insomnia is one of the most common sleep disorders with a prevalence of ten percent of the general adult population in western industrial countries [1], and it is accompanied by reduced quality of life, high socio-economic costs, as well as an elevated risk for physical [2] and mental [3] morbidity.

A central aspect of current pathophysiological concepts of chronic insomnia disorder is central nervous hyperarousal [4,5]. It is postulated that psychological mechanisms such as enhanced sleep reactivity to stress [6] and excessive negatively toned cognitive activity [7] as well as over-active neurobiological systems such as the humoral stress system [8], or deficient inhibitory systems such as reduced cortical GABA transmission [9] as a final common pathway result in heightened emotional, cognitive and physiological arousal interfering with sleep. Data from imaging studies, although heterogenous, are mostly discussed as supporting the concept of hyperarousal—for a review, see [10]. Regarding electroencephalographic measures of cortical arousal in insomnia, there is ample evidence for hyperarousal during night time, in non-rapid eye movement (NREM) sleep [11,12], rapid eye movement (REM) sleep [13], or at sleep onset [14].

These studies referring to sleep or the transition from waking state to sleep leave open whether enhanced arousal may occur specifically in the sleep context, e.g., as a result of a conditioned physiological reaction to sleep-related stimuli [15] or whether insomnia also involves daytime hyperarousal. As a clinical observation, preserved daytime alertness in spite of sleep disturbance is a typical feature of insomnia disorder [16]. Studies using the multiple sleep latency test (MSLT) as a measure of daytime sleep propensity reported enhanced sleep latency during daytime in insomnia patients [17,18], with the highest sleep latencies occurring in those patients showing the shortest total sleep time during the night [19]. However, in view of the instructions for the MSLT procedure, namely, to try to fall asleep, this finding may also refer to sleep context-related mechanisms. In contrast, several electroencephalography (EEG) studies during the wake resting state investigated EEG spectral power in insomnia patients and found globally [20] or frontally [21,22] enhanced beta power, thus consistent with cortical hyperarousal. Data concerning this, however, are inconsistent [23].

Another perspective on daytime regulation of arousal and vigilance is the evolution of fine-grained vigilance stages over time during the resting state. To this purpose, the working group of U. Hegerl in Leipzig developed an EEG paradigm called Vigilance Algorithm Leipzig (VIGALL) [24]. VIGALL allows an objective classification of seven vigilance stages ranging from wakefulness to sleep and their evolution over time and has been validated by simultaneous functional magnetic resonance imaging (fMRI) (Olbrich, et al., 2009) and positron emission tomography (PET) analyses (Guenther, et al., 2011) as well as against the Multiple Sleep Latency Test (Olbrich, et al., 2015). Investigation of patients with depression using this paradigm revealed a state of arousal hyperstability compared to healthy controls, i.e., during resting-state conditions depressive patients remain in higher vigilance stages [25] and arousal regulation has been investigated as a possible predictor to antidepressants (Schmidt et al., 2017) and sleep deprivation therapy (Sander et al., 2019).

Depression and insomnia disorder share clinical aspects (cognitive arousal and sleep disturbance), neurobiological features (over-activation of the humoral stress system [8] and disordered arousal) and epidemiological characteristics [3]. Thus, an interesting question is whether both disorders share daytime vigilance hyperstability as measured by the VIGALL. If so, disordered arousal regulation might constitute a common link between insomnia disorder and depression in the sense of a transdiagnostic neurobiological dimension (Research Domain Criteria project, RDoC) [26] predisposing individuals to both disorders and thereby contributing to their epidemiological overlap [3]. Interestingly, two recent genome-wide association (GWA) studies point to a possible link between vigilance regulation [27] or complaints of insomnia [28] on the one hand and depressive symptoms on the other hand, supporting the concept of a transdiagnostic arousal disorder.

Thus, in a first step, we aimed at analyzing resting-state vigilance stability in patients with insomnia disorder. We hypothesized that patients with insomnia show a more stable arousal regulation during daytime as measured by the VIGALL arousal stability score (ASS), i.e., that they show an increased amount of higher vigilance stages or their slower decline over time compared to healthy subjects. We also hypothesized that enhanced arousal stability in insomnia is correlated to measures of objective sleep disturbance.

Furthermore, there is increasing evidence that chronic insomnia may not be a uniform disorder but that clinically definable subgroups can be distinguished. In this context, insomnia with objective short sleep duration was reported to be a biologically more severe phenotype involving activation of both limbs of the stress system, cortical hyperarousal, higher somatic morbidity risk and a stronger genetic background [29]. Thus, in an additional exploratory analysis, we aimed at analyzing whether daytime vigilance stability in insomnia differs between subgroups characterized by different extents of objective sleep disturbance.

## 2. Materials and Methods

### 2.1. Study Participants

We investigated a total of 72 participants aged between 18 and 65 years comprising 41 unmedicated patients with a current ICSD-3 (International Classification of Sleep Disorders, 3rd edition) diagnosis of chronic insomnia assessed by a clinician certified in sleep medicine (CS) and 31 healthy volunteers not suffering from any sleep or neuropsychiatric disorder. Insomnia patients had to be free of any other sleep disorder and any neuropsychiatric disorder. Further exclusion criteria for both groups were deviance of the sleep wake rhythm, shift work including night shift, recent travel across time zones, any serious physical disease as well as any intake of psychotropic medication. Patients and controls were required not to substantially deviate from their normal sleep–wake rhythm prior to the study. Both groups did not differ with regard to circadian rhythm preference as indicated by the Morningness–Eveningness Questionnaire, German version (D-MEQ, Table 1). Insomnia patients were recruited through the outpatient clinic of the sleep laboratory of the Central Institute of Mental Health, whereas healthy volunteers were recruited through our homepage. Five subjects (three patients and two controls) had to be excluded from analysis due to technical issues (low voltage EEG incompatible with the application of the EEG algorithm). Furthermore, eight subjects (four patients and four controls) had to be excluded due to polysomnographic evidence for organic sleep disorders not diagnosed by sleep history (periodic limb movements during sleep (PLMS) with PLMS index exceeding15/h or sleep apnea syndrome with apnea hypopnea index (AHI) of 10/h or more. Thus, the final sample consisted of 34 insomnia patients and 25 healthy controls. The two groups did not differ in regard to age and sex (see Table 1).

### 2.2. Procedures

Participants underwent a twenty-minute resting-state electroencephalography (EEG) with eyes closed in the sleep laboratory of the Central Institute of Mental Health in Mannheim, Germany. All measurements took place in the same time frame between 08:30 and 10:30 a.m. in order to exclude different circadian effects on arousal. During the night preceding the resting-state EEG, participants underwent a polysomnography (PSG) with standardized bedtimes from 11 p.m. (lights off) to 6:30 a.m. (lights on) in close agreement with the subjects’ normal rhythm. The aim of PSG recording was to rule out any undiagnosed sleep disorders and to be able to control for the effect of sleep quality on arousal stability the next day. Polysomnographic investigation of objective sleep parameters was complemented by the assessment of subjective insomnia severity and psychometric measures (see psychometric measures section).

The study protocol was approved by the ethics committee II of the Medical Faculty Mannheim of the University of Heidelberg (2016-562N-MA), Medical Faculty Mannheim, following the rules of the Declaration of Helsinki of 1975, revised in 2013 [30]. All participants were informed about the study aims and procedures and gave their written consent prior to the investigation.

### 2.3. Resting-State EEG Measurement

The measurements took place in a sound-attenuated and dimly lit room where participants were comfortably seated in half-reclined position. Consumption of caffeine and nicotine was prohibited prior to the measurement. The resting-state EEG was recorded with 19 Ag/AgCl electrodes (10–20 international system of EEG electrodes placement, Easy-Cap GmbH, Herrsching, Germany) using the common average as reference. Electrode impedances were kept below 10 kΩ if possible. Additionally, electrooculogram (EOG) and electrocardiogram (EKG) were recorded. EEG signals were recorded with a polysomnograph (Nihon Kohden EEG1212/PSG) at a sampling rate of 200 Hz.

Initially, participants were asked to close and open their eyes (Berger maneuver) in order to assess their individual alpha-rhythm. This was followed by a simple arithmetic task (consecutively subtracting 6 from 100) in order to ensure that all participants started the 20-min resting-state EEG in a comparably activated state of arousal. Finally, at the onset of the 20-min resting EEG, participants were reminded to neither try to fall asleep nor to forcefully stay awake, but to relax during the measurement.

### 2.4. Polysomnography

Polysomnography was performed according to the criteria of the American Academy of Sleep Medicine [31] including EEG in seven derivations (F4–A1, C4–A1, O2–A1, Cz-A1, F3–A2, C3–A2 and O1–A2), surface electromyography (EMG) of the chin and both tibialis anterior muscles, two-sided electrooculography (EOG), electrocardiography (ECG) and recording of respiratory variables. We used a Nihon Kohden EEG1212/PSG polysomnograph and a sampling rate of 200 Hz. Prior to and during polysomnography, consumption of nicotine and caffeine was prohibited. Sleep stage classification and detection of artefacts and arousals for each 30 s epoch were performed visually by an experienced rater according to the criteria of the American Academy of Sleep Medicine [31].

### 2.5. Psychometric Measures

Subjective insomnia severity was assessed with the Insomnia Severity Index (ISI) [32] and the Pittsburgh Sleep Quality Index (PSQI) [33]. Trait arousal was measured using the Arousal Predisposition Scale (APS) [34], whereas the Pre-sleep Arousal Scale (PSAS) [35] was used to assess state cognitive and somatic arousal. Daytime sleepiness was measured with the Epworth Sleepiness Scale (ESS) [36] and subjective sleepiness directly before and after the resting-state EEG measurement with the Karolinska Sleepiness Scale (KSS, [37]). Sleep-related metacognitive beliefs were assessed using the German translation of the Metacognitions Questionnaire-Insomnia (MCQ-I) [38], stress reactivity with the Perceived Stress Reactivity Scale (PSRS-23) [39] and depressiveness with the Beck Depression Inventory (BDI) [40]. The two sleep/wake-related items of the BDI (items 16 and 17) were not included in the sum score in order to avoid the measure to be biased by the sleep disorder. Due to technical errors, about half of the participants only completed the first page of the BDI (items 1–10). However, due to the high internal consistency of the test, both versions (items 1–10 and items 1–21 without sleep items) were highly intercorrelated (Pearson correlation r = 0.945, *p* < 0.0001 for *N* = 33 patients). Thus, we used the 10-item score for further analyses. Circadian rhythm preference was assessed using the German version of the Morningness–Eveningness Questionnaire (D-MEQ) [41].

### 2.6. EEG Vigilance Classification Using VIGALL

EEG pre-processing was performed with the software package BrainVision Analyzer 2 (Brain Products GmbH, Munich, Germany) according to the procedures described elsewhere [42]. The preprocessing (performed by authors A.L. and C.S. (Christian Sander)) included digital filtering (high pass: 0.5 Hz, low pass: 70 Hz, notch: 50 Hz), segmentation to 1 s intervals, screening and exclusion of segments containing temporally circumscribed artifacts. Cardioballistic, eye movement or persistent muscle artifacts were corrected using an independent component analysis (ICA)-based approach. Subsequently, the original 19 EEG channels were expanded to the 25-channel VIGALL standard using channel interpolation. In parallel, K-complexes and sleep spindles were manually identified and marked (by author C.S. (Claudia Schilling)). Afterwards, vigilance classification was performed using VIGALL 2.1. This semi-automatic computer algorithm allows allocation of 1 s EEG epochs to 1 of 7 EEG vigilance stages (for detailed descriptions of the scoring algorithm, see Sander et al. [24]:

- Stage 0 representing an activated wake state as seen in mental effort. This stage is recognized by a low amplitude EEG with non-alpha-activity, typically without the presence of slow horizontal eye movements.
- Stage A represents relaxed wakefulness: The EEG is dominated by prominent alpha activity. Stage A can be subdivided into A1, A2 and A3, according to the degrees of frontalization of alpha activity from occipital to anterior brain regions. A predominant occipital alpha-activity characterizes stage A1, while alpha-activity shifts to frontal and temporal regions in A2/3 stages accompanied by a decrease in amplitude.
- Stage B corresponds to drowsiness. This stage can be divided into sub-stages B1 (characterized by low amplitude EEG without alpha-activity, with the presence of slow horizontal eye movements) and B2/3 (dominated by theta and/or delta activity).
- Stage C reflects sleep onset. Since this stage is characterized by sleep spindles and K-complexes, its classification is bound to the manually set markers of these EEG phenomena in EEG epochs of 1 s. Spindles and K-complexes were identified according to the criteria of the American Academy of Sleep Medicine [31] by an experienced rater certified in sleep medicine.

In the next step, the VIGALL classification results were imported into a customized Excel template with Visual Basic for Applications (VBA) macros (Microsoft). This template was used to compute several vigilance parameters:

(a) For each recording minute as well as the total recording period, the absolute amount of vigilance stages (0, A1, A2/3, B1, B2/3 and C) was counted, and the percentage amount was calculated (amount ∗ 100/total number of non-artefact segments).
(b) Furthermore, each EEG vigilance stage is assigned a numerical score ranging from 1–7 (C = 1, B2/3 = 2, B1 = 3, A3 = 4, A2 = 5, A1 = 6, 0 = 7). Again for each recording minute as well as the total recording, a mean vigilance value (MVV) was calculated by averaging the scores of all non-artefact segments.
(c) In addition, an arousal stability score (ASS) was determined for each subject’s EEG vigilance time course to quantify the speed and extent of the respective decline in brain arousal. For this, successive blocks with a duration of 1 min were analyzed concerning fulfilment of one of the following criteria: (I) at least 2/3 of all segments classified as 0/A or 0/A1 stages; (II) at least 1/3 of all segments classified as B stages (B1 + B2/3); (III) at least 1/3 of all segments classified as B2/3 stages; (IV) occurrence of at least 1 C stage. The 20-min EEG recording was separated into four consecutive 5-min epochs (quartiles). If only criterion I was fulfilled during the whole recording, a high ASS-score is given (14 (only 0/A1 stages) or 13 (only 0/A-stages)). Depending on which of the criteria II to IV were achieved in which quarter of the measurement, increasingly lower ASS scores were awarded (e.g., C stages reached within the last quarter: ASS = 4, C-stages reached within the first quarter: ASS = 1). Thus, higher ASS scores correspond to higher arousal stability.
(d) Individual time courses were compared with prototypical time courses corresponding to the three prototypical types of brain arousal regulation according the model of Hegerl et al. [43]: adaptive vigilance regulation (graduated decrease in vigilance over time), unstable vigilance regulation (accelerated decrease in vigilance) and (hyper) stable vigilance regulation (absence of vigilance decrease). Each participant was assigned to the prototype course with the smallest sum of deviation squares. Post hoc, an additional model distinguishing four prototypes of brain arousal regulation was calculated. In this model, in addition to the adaptive and the unstable regulatory type, a stable (no vigilance decrease over time) and a hyperstable regulatory (retention in the highest arousal states) type were distinguished.

### 2.7. Statistical Analysis

Statistical analyses were performed using the Statistical Package for Social Sciences version 25 (IBM SPSS, Inc., Chicago, IL, USA). Group differences in demographic, sleep and psychometric parameters were analyzed using *t*-tests and chi-square tests. For parametric variables that are not normally distributed, non-parametric tests were applied, e.g., the Mann–Whitney U test for the arousal stability score. Directed hypotheses were tested one-tailed. The significance level was set at α = 0.05.

In order to exploratively test the hypothesis that arousal regulation may differ between insomnia subgroups characterized by different degrees in objective sleep disturbance, we defined insomnia subgroups by means of a median split with regard to sleep efficiency (i.e., at a sleep efficiency of 76.5%).

## 3. Results

### 3.1. Group Differences in Polysomnographic and Psychometric Measures

As expected, insomnia patients but not healthy controls showed enhanced scores for subjective insomnia severity (Table 1). Furthermore, both groups differed significantly in a variety of PSG parameters reflecting the insomnia disorder. Furthermore, psychometric variables relating to stress reactivity, state and trait arousal, and insomnia-related metacognitions also showed strong group differences in the expected direction. Lastly, insomnia patients scored significantly higher on self-rated depressive symptoms (Table 1).

### 3.2. Daytime Vigilance Stability

**Figure 1:**
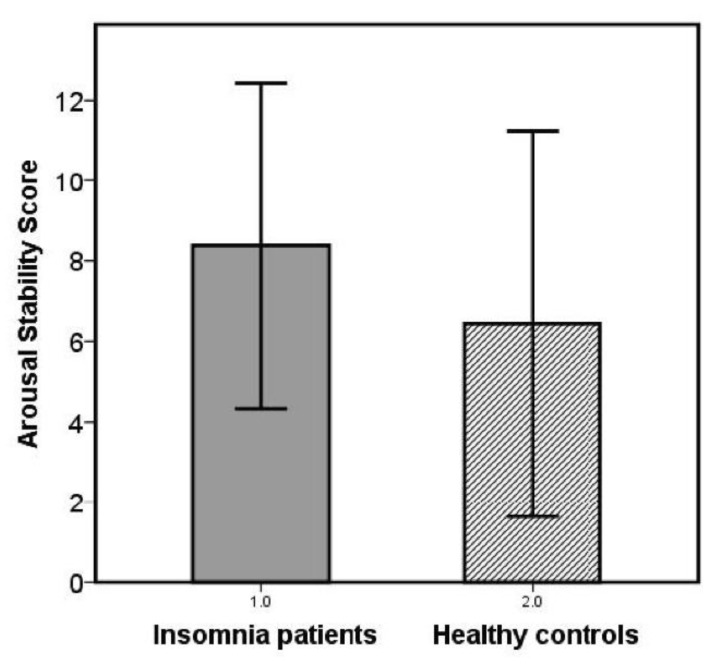
Arousal stability scores in patients with insomnia disorder and healthy control subjects.

**Figure 2:**
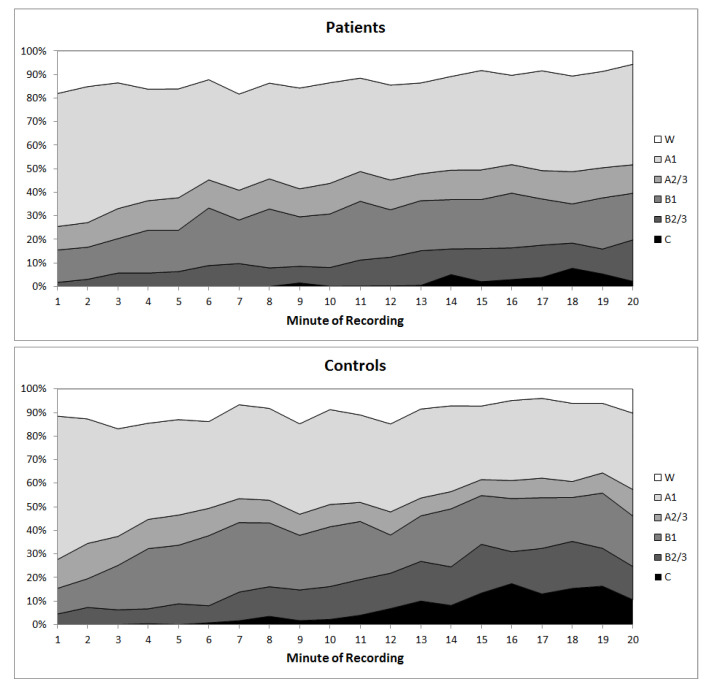
Time-course of EEG arousal stages across the 20-min resting EEG in insomnia patients (above) and healthy controls (below).

Patients showed a less pronounced decline in vigilance stages over the 20-min time interval compared to healthy controls resulting in a significantly higher arousal stability score (Mann–Whitney U test; U = 314.5; R = −1.7; *p* = 0.044, one-tailed, Figure 1 and Figure 2).

Arousal stability scores did not correlate with polysomnographic sleep parameters in the total insomnia sample nor in healthy subjects (Table 2). Moreover, self-rated depressiveness did not correlate with the arousal stability score in insomnia patients nor in healthy subjects (Table 2).

Explorative analysis of differences in vigilance sub-states revealed that C-stages occurred significantly less frequent in insomnia patients compared to controls (insomnia patients: occurrence in 10/34 patients, mean number of C-stages = 18.6 ± 47.3; controls: occurrence in 14/25 subjects, mean number = 74.1 ± 111.3; t = −2.3; *p* = 0.026, two-tailed, Figure 2).

Regarding subjective vigilance data (KSS score), insomnia patients felt sleepier before the resting-state EEG session compared to controls (Table 1), but nevertheless showed more stable vigilance regulation than healthy controls (see above). Subjective data on change in sleepiness from the pre to post resting-state EEG paralleled objective vigilance data, i.e., the arousal stability score, as insomnia patients reported no change in sleepiness (KSS diff), whereas healthy controls reported a slight increasing in sleepiness (Table 1).

**Figure 3:**
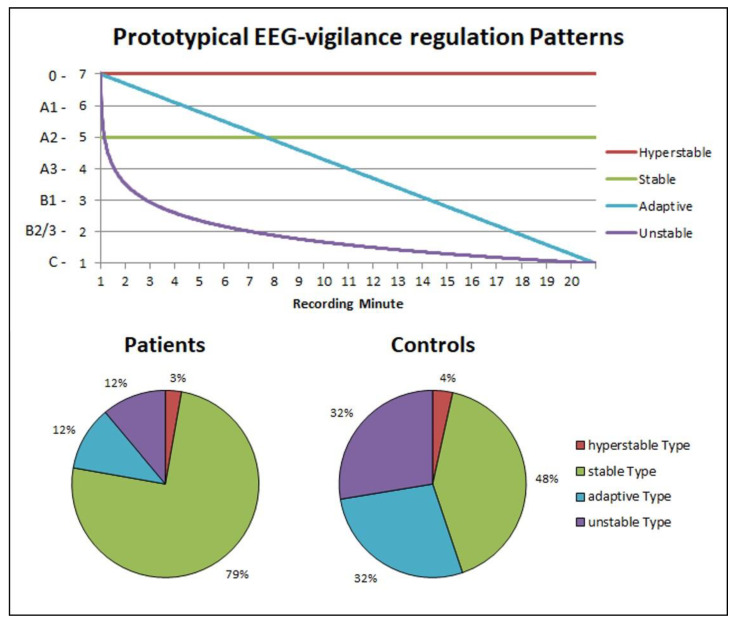
Frequency of prototypical vigilance regulation time courses. Differential distribution of prototypes: chi-square test; *p* = 0.044 (one-tailed).

Comparison of individual time courses according to the model of Hegerl et al. [25] distinguishing three prototypes of brain arousal regulation did not reveal significant differences between insomnia patients and controls (X^2^ = 2.138; *p* = 0.172, one-tailed). However, explorative modeling of a four prototype solution better distinguished between both groups. Consistent with the hypothesis of a more stable arousal regulation in insomnia, this model revealed a markedly higher proportion of insomnia patients in the “stable” cluster at the expense of the “adaptive” and “unstable” clusters (X^2^ = 6.549; *p* = 0.044, one-tailed, Figure 3).

### 3.3. Insomnia Subtypes with Respect to the Extent of Objective Sleep Disturbance

The characteristics of insomnia subgroups with a sleep efficiency above and below the median split of 76.5% are given in Table 3. The ASS of the high sleep efficiency group was significantly higher than that of the healthy control group (N = 17 vs. 25; Mann–Whitney U test: U = 142.0; R = −1.8; *p* = 0.035, one-tailed), whereas the arousal stability score of the low sleep efficiency group did not differ from controls (N = 17 vs. 25; Mann–Whitney U test: U = 172.5; R = −1.0; *p* = 0.151, one-tailed).

**Figure 4:**
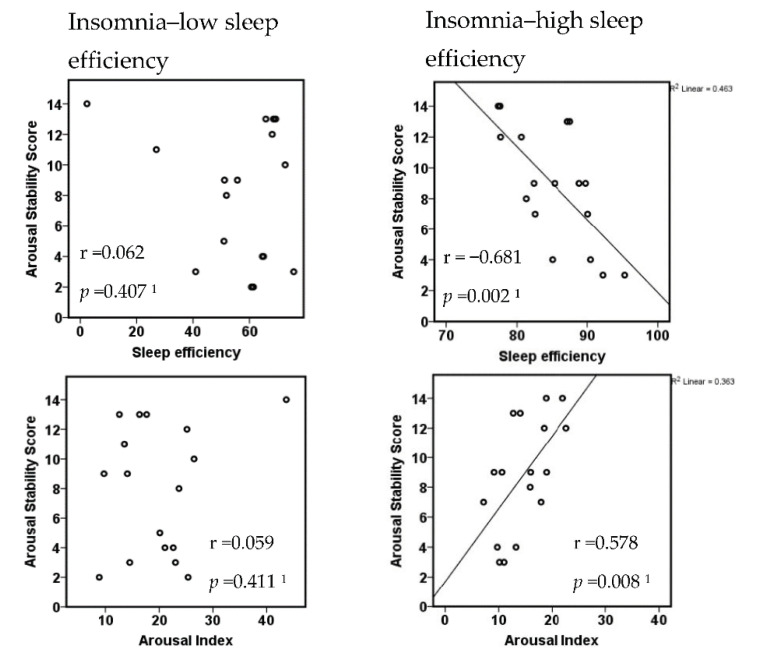
Correlation of the arousal stability score with polysomnographic sleep measures in insomnia subgroups with high versus low sleep efficiency. ^1^ Spearman correlation, one-sided testing corresponding to directed hypothesis.

The two subgroups divided regarding their objective sleep quality did neither differ in subjective insomnia severity (ISI, PSQI) nor in psychometric characteristics (see Table 3). However, they differed with respect to the correlation between the ASS and measures of objective sleep disturbance. While patients with higher sleep efficiency showed strong and highly significant correlations between ASS and sleep efficiency (negative correlation) or arousal index (positive correlation), patients with low sleep efficiency did not (Figure 4).

**Figure 5:**
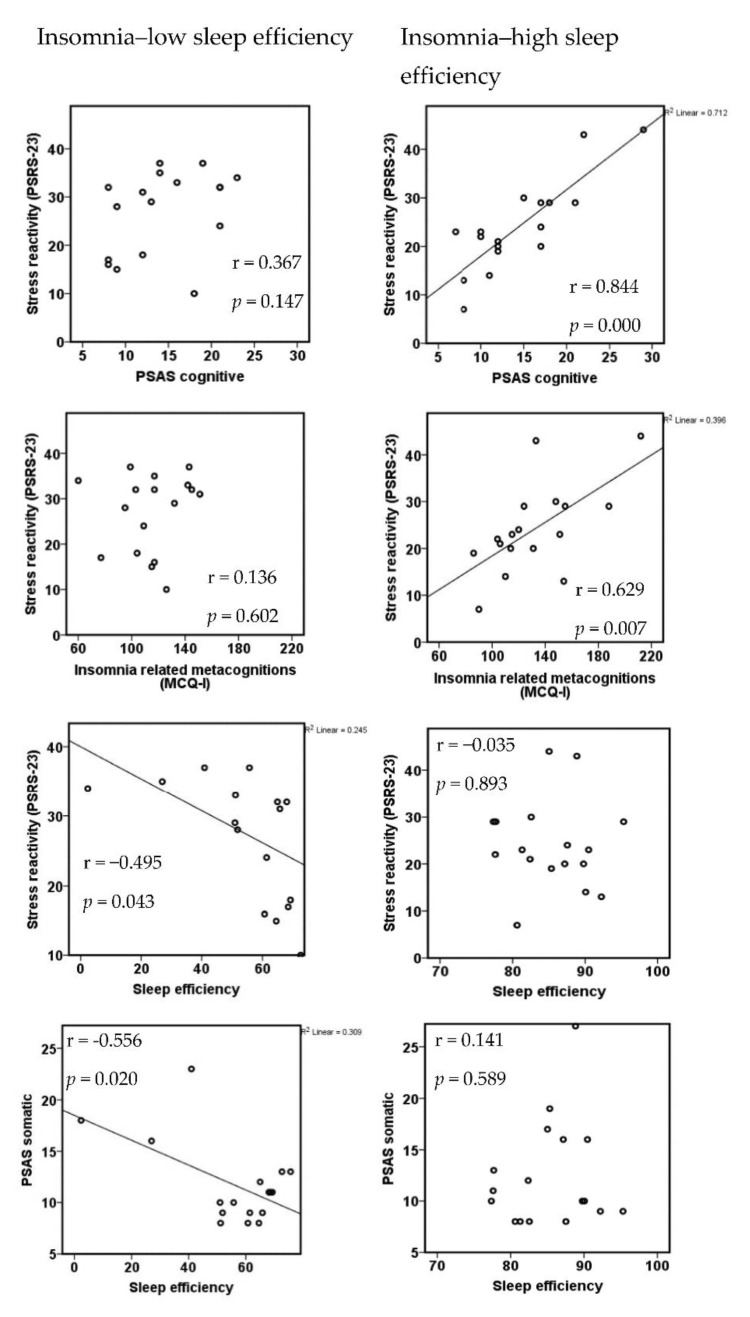
Different psychophysiological mechanisms in insomnia subgroups with high versus low sleep efficiency.

Further explorative analysis of psychophysiological correlates of arousal regulation in both subgroups revealed the following: insomnia patients with higher sleep efficiency (less objective sleep disturbance) showed strong correlations between stress reactivity (PSRS-23) and state cognitive arousal (PSAS cognitive subscale) as well as insomnia-related cognitions and metacognitions (MCQ-I, Figure 5). Furthermore, in these patients, stress reactivity strongly correlated with trait arousal (APS, r = 0.823; *p* = 0.000), but not with sleep measures such as sleep efficiency (r = −0.035; *p* = 0.893), arousal index (r = 0.080; *p* = 0.760) or wake after sleep onset (WASO; r = 0.221; *p* = 0.394). In contrast, patients with lower sleep efficiency (more severe objective sleep disturbance) did not show correlations between stress reactivity and state cognitive arousal or insomnia-related cognitions and metacognitions but strong negative correlations of objective sleep parameters (sleep efficiency) with somatic arousal (PSAS somatic subscale) and stress reactivity (Figure 5), as well as with subjective sleepiness (KSS, r = −0.611; *p* = 0.009).

## 4. Discussion

In agreement with our hypothesis, we found (1) hyperstable vigilance regulation during daytime in patients with insomnia disorder, i.e., insomnia patients during resting-state conditions, showed a less pronounced decline in vigilance stages over a 20-min time interval compared to healthy control subjects. (2) Increased vigilance stability correlated with polysomnographic measures of reduced sleep quality in insomnia patients with high to moderately reduced sleep efficiency. (3) Explorative analysis revealed that group differences in vigilance stability were mainly due to different numbers of spindles and K-complexes. (4) Subgroup analyses indicated that vigilance stability was especially pronounced in insomnia patients with mildly reduced sleep efficiency (PSG measure), and both groups with mildly reduced and markedly reduced sleep efficiency showed different correlation patterns between psychometric variables and vigilance regulation.

Our findings indicate that there is a daytime hyperarousal in insomnia disorder independently of the sleep context. Our vigilance measurement during the resting state did not include an instruction to try to fall asleep (like typically the MSLT protocol [44]), and, thus, enhanced vigilance stability cannot be attributed to sleep effort or conditioned arousal typically found in primary insomnia patients at sleep onset. This is in accordance with the previous EEG resting state [20,21,22]. However, there are studies that are not able to demonstrate a hyperarousal during wakefulness [23]. Our data also provide a temporal perspective in the decline of vigilance stages over 20 min. The temporal perspective allowed a classification of insomnia patients and controls into distinct types with specific brain arousal regulation: insomnia patients predominantly map to the stable type of arousal regulation, whereas healthy participants showed a gradual vigilance decline.

We expected increased daytime arousal stability in insomnia patients to be correlated with polysomnographic markers of objective sleep disturbance. Although this was not the case in the total insomnia sample, the insomnia patient subgroup with mildly reduced sleep efficiency (sleep efficiency above 76.5%) did show strong correlations between daytime arousal stability and disturbed sleep continuity as well as arousal index. In the insomnia subgroup with severely reduced sleep efficiency, in contrast, daytime vigilance stability probably was affected by increased sleep pressure due to marked sleep loss (mean sleep duration during the preceding night: 260 min). Subjective vigilance ratings (KSS) prior and after the test session paralleled the EEG finding of hyperstable arousal regulation in insomnia. Thus, in insomnia disorder heightened arousal regulation includes the night as well as the day, in line with the previously postulated concept of a 24 h hyperarousal, suggesting insomnia rather being a disorder of arousal and not restricted to sleep complaints [4,5,45].

In contrast to the polysomnographic sleep parameters, none of the psychometric parameters including self-rated depressive mood correlated with the arousal stability score, neither in insomnia patients nor in healthy subjects. This is important to note, as self-rated depressive symptoms differed between insomnia patients and healthy controls and thus could have been viewed as a potential confounder regarding enhanced arousal in insomnia disorder. Vigilance hyperstability using the VIGALL algorithm has also been demonstrated in patients suffering from depression [25]. Thus, enhanced vigilance stability links both disorders to disturbed arousal regulation, in accordance with the concept of disordered arousal as a transdiagnostic feature [26]. However, subclinical mood disturbances (in contrast to a diagnosis of a major depressive disorder) seem not to affect vigilance regulation during the day in a marked way.

A closer look at the hyperstable arousal regulation in both disorders reveals that depressive patients remained throughout the 20-min time period in high vigilance stages (stage A1) [25], whereas the insomnia patients in our study declined into lower vigilance stages (B2/3) but did not pass into light sleep as in the case of some of the healthy controls. This might reflect different neurophysiological mechanisms behind hyperarousal in depression and in insomnia disorder, or a more pronounced disturbance in depression compared to primary insomnia. In our study, group differences in arousal stability scores were partly explained by reduced frequencies of spindles and K-complexes in the insomnia group. Thus, our findings corroborate recent reports of reduced sleep spindle activity [46,47] and K-complexes [48] and instability of stage N2 sleep [49,50] in insomnia patients. These findings thus also occur at the transition from wake to sleep. However, other studies did not show a reduction of these events [51,52]. This might point to the fact that insomnia disorder does not represent a homogeneous disease entity.

Insomnia with and without objectively disordered sleep (polysomnographically determined sleep efficiency) may constitute two distinct subtypes differing in regard to their biological background, namely, risk of comorbidity and presence of physiological hyperarousal, e.g., hypercortisolemia only found in patients with reduced sleep efficiency [29,53]. Our explorative investigation of two insomnia subgroups characterized by high and low sleep efficiency revealed that vigilance stability was significantly enhanced particularly in the high sleep efficiency group, i.e., in those insomnia patients showing only mild sleep disturbances according to standard sleep parameters. Moreover, in these patients, arousal stability strongly correlated with several parameters of disturbed sleep continuity and arousal, whereas in patients with markedly reduced sleep efficiency, this correlation presumably was overridden by the effect of increased sleep pressure.

In insomnia with high sleep efficiency, stress reactivity strongly correlated with trait arousal, state cognitive arousal and insomnia-related cognitions and metacognitions, in line with current models of insomnia [54,55,56], but not with sleep parameters. On the other hand, patients with low sleep efficiency showed strong relationships between objective sleep disturbance and somatic arousal, stress reactivity and subjective sleepiness (KSS score). Thus, our findings support the idea of distinctive subgroups within the insomnia disorder, but our subgroup classification does not match subgroups recently described by Blanken et al. [57], as different classification criteria were used (polysomnographic sleep data in our study versus psychometric data in the study of Blanken et al.).

A clear strength of our study is that insomnia patients and controls were characterized by a full sleep laboratory procedure, ensuring the exclusion of comorbidities, periodic limb movement disorder, and the opportunity to relate polysomnographic and psychometric measures with vigilance regulation. Due to strict exclusion criteria, i.e., excluding any psychiatric comorbidity and any other sleep disorder like restless legs syndrome or periodic limb movements during sleep, our patient sample consists of the subtype psychophysiological or primary insomnia (cf. ICSD-2) with an etiology focusing on hyper-arousal and sleep-related thoughts as key symptoms. A further strength is that the VIGALL algorithm provides a temporal perspective (20-min time course) compared to previous daytime arousal investigations in insomnia [20,21,22] and, thus, enables additional insights into potentially implicated mechanism of vigilance regulation.

One limitation of the present study is that the patient sample may not be a representative for all insomnia patients, as patients seeking help in a specialized sleep clinic might be more severely affected. However, the differentiation in subtypes with mild sleep impairment and marked sleep impairment indicates that we might have included the whole range of insomnia severity in our study.

In conclusion, measuring vigilance dynamics during resting state revealed hyperstable vigilance regulation during the day in insomnia disorder as a manifestation of daytime hyperarousal. This was found particularly in insomnia patients with only mild reduction in polysomnographically measured sleep efficiency. Characterization of different facets of EEG hyperarousal (i.e., during waking and sleep) may enhance our understanding of the implicated mechanisms. A better understanding of pathophysiological mechanisms and differentiation of insomnia subtypes is essential for the development of biomarkers for treatment choice and outcome in view of a personalized sleep medicine. Future studies should investigate the link behind disordered arousal regulation and the phasic components of stage N2 sleep. In addition, it would be interesting to study how the concept of local sleep [12] or superficial sleep bouts [50] during definite sleep in insomnia is related to mechanisms of vigilance regulation. A clinically important question is whether hyperstable arousal regulation in insomnia can be reversed by effective therapy or whether it represents a trait characteristic.

## Figures and Tables

**Table 1:** Demographic and psychometric characteristics and polysomnographic sleep parameters.

	Insomnia (*n* = 34)	Healthy Controls (*n* = 25)	t	*p*
Demographics				
Age (years)	44.1 ± 12.5	39.2 ± 13.0	1.5	0.153
Sex (w/m)	27/7	19/6	0.1 ^1^	0.755
Psychometric data				
Disturbed sleep quality (PSQI)	11.9 ± 3.1 ^2^	4.3 ± 2.3	9.9	0.000 *
Insomnia severity (ISI)	17.5 ± 4.3 ^3^	3.0 ± 3.1 ^4^	14.2	0.000 *
Sleep-related beliefs (MCQ-I)	123.3 ± 30.1	98.4 ± 21.4	3.5	0.001 *
Stress reactivity (PSRS-23)	25.6 ± 9.1	16.7 ± 5.5	4.7	0.000 *
Trait arousal (APS)	33.9 ± 6.2	28.4 ± 4.3	3.8	0.000 *
Sleepiness (ESS)	7.0 ± 5.2	7.2 ± 4.3	−0.2	0.876
Depressiveness (BDI 1–10)	3.2 ± 3.3 ^2^	1.2 ± 1.8	2.7	0.010 *
Morningness–Eveningness (D-MEQ)	56.0 ± 8.2 ^5^	54.6 ± 9.1	0.6	0.544
State arousal (PSAS)				
Arousal	26.6 ± 8.6	18.3 ± 3.1	4.6	0.000 *
Somatic arousal	12.1 ± 4.6	8.8 ± 1.2	4.0	0.000 *
Cognitive arousal	14.5 ± 5.5	9.5 ± 2.5	4.6	0.000 *
State Sleepiness (KSS)				
Pre EEG	5.3 ± 1.7	3.5 ± 1.9	3.8	0.000 *
Post EEG	5.3 ± 2.0	4.7 ± 2.1	1.1	0.274
pre–post diff	0.0 ± 2.4	1.2 ± 2.8	−1.8	0.085 ^(^*^)^
Sleep parameters				
Sleep latency (min)	31.9 ± 34.2	25.6 ± 17.8	0.8	0.405
Sleep efficiency (%)	70.7 ± 20.1	83.6 ± 12.9	−3.0	0.004 *
WASO (min)	91.7 ± 63.9	42.9 ± 42.8	3.5	0.001 *
Total sleep time (min)	323 ± 92	382 ± 58	−3.0	0.004 *
% Stage 1	14.2 ± 5.8	12.2 ± 5.2	1.4	0.171
% Stage 2	38.4 ± 11.3	43.9 ± 8.7	−2.0	0.051 ^(^*^)^
% Stage 3	13.4 ± 7.4	20.8 ± 9.7	−3.3	0.001 *
% REM	10.5 ± 5.1	12.7 ± 5.4	−1.6	0.125
Arousal index	17.3 ± 7.1	14.0 ± 8.0	1.7	0.104
Number of wake periods	29.8 ± 13.4	23.8 ± 12.0	1.8	0.081 ^(^*^)^

Data reported as mean ± standard deviation; ^2^ N = 27; ^3^ N = 32; ^4^ N = 24; ^5^ N = 31. ^1^ Chi-square test (all other parameters *t*-test); * significance at *p* ≤ 0.05; (*) trend level significance at *p* ≤ 0.1. PSQI = Pittsburgh Sleep Index; ISI = Insomnia Severity Index; MCQ-I = Metacognitions Questionnaire-Insomnia; PSRS-23 = Perceived Stress Reactivity Scale; APS = Arousal Predisposition Scale; ESS = Epworth Sleepiness Questionnaire; BDI 1–10 = Beck Depression Inventory (items 1–10); D-MEQ = Morningness–Eveningness Questionnaire, German version; PSAS = Pre-sleep Arousal Scale; KSS = Karolinska Sleepiness Scale; EEG = electroencephalography; WASO = Wake after sleep onset.

**Table 2:** Correlations of the Arousal stability score with subjective and objective sleep parameters and psychometric measures.

	Insomnia (*n* = 34)		Healthy Subjects (*n* = 25)	
	r	*p*	r	*p*
Subjective sleep				
Insomnia severity (ISI)	−0.116 ^1^	0.529	0.009 ^2^	0.968
Objective sleep				
Sleep efficiency (SE)	−0.058	0.372 ^3^	−0.064	0.761
Arousal index (AI)	0.199	0.129 ^3^	−0.106	0.614
Psychometric parameters				
Stress reactivity (PSRS-23)	−0.035	0.846	−0.093	0.660
Trait arousal (APS)	0.075	0.671	0.058	0.784
State arousal (PSAS)				
Arousal	0.135	0.447	−0.139	0.507
Somatic arousal	0.145	0.414	−0.121	0.565
Cognitive arousal	0.148	0.403	0.124	−0.554
Insomnia-related beliefs (MCQ-I)	−0.205	0.244	0.045	0.836
Depressiveness (BDI 1–10)	−0.060 ^1^	0.745	0.236	0.257

^1^ N = 32; ^2^ N = 24; Spearman correlation, ^3^ one-sided testing.

**Table 3:** Insomnia subgroups with respect to the degree of objective sleep disturbance.

	Low Sleep Efficiency (*n* = 17)	High Sleep Efficiency (*n* = 17)	t	*p*
Arousal Stability Score	7.9 ± 4.4	8.8 ± 3.8	0.6 ^1^	0.534
Subjective insomnia severity				
Disturbed sleep quality (PSQI)	11.7 ± 3.2	12.1 ± 3.0	0.3	0.755
Insomnia severity (ISI)	17.4 ± 3.9	17.7 ± 4.8	0.2	0.805
Psychometrics				
Insomnia-related beliefs (MCQ-I)	114.8 ± 24.4	131.8 ± 33.4	2.9	0.100
Depressiveness (BDI 1–10)	2.5 ± 2.3	4.0 ± 4.0	1.3	0.191
Stress reactivity (PSRS-23)	27.1 ± 8.6	24.1 ± 9.5	−0.9	0.352
Sleepiness (ESS)	6.4 ± 5.5	7.9 ± 5.0	0.7	0.520
Trait arousal (APS)	34.2 ± 5.1	33.6 ± 7.2	−0.3	0.786
State sleepiness (KSS)				
pre EEG	5.8 ± 1.5	4.8 ± 1.9	−1.7	0.095 ^(^*^)^
post EEG	5.4 ± 1.7	5.1 ± 2.3	−0.4	0.671
pre post diff	−0.4 ± 1.8	0.4 ± 3.0	0.8	0.410
State Arousal (PSAS)				
Arousal	26.2 ± 7.9	26.9 ± 9.4	0.2	0.830
Somatic arousal	11.7 ± 4.0	12.4 ± 5.1	0.4	0.659
Cognitive arousal	14.5 ± 5.2	14.5 ± 5.9	0.0	1.00
History of previous depressive episode	6/17	3/17	1.4 ^2^	0.244

*t*-tests except for ^1^ Mann–Whitney U test (U), ^2^ chi-square test; ^(^*^)^ trend level significance (*p* ≤ 0.1).

## References

[1] Ohayon M.M. (2002). Epidemiology of insomnia: What we know and what we still need to learn. Sleep Med. Rev..

[2] Leger D., Guilleminault C., Bader G., Levy E., Paillard M. (2002). Medical and socio-professional impact of insomnia. Sleep.

[3] Baglioni C., Battagliese G., Feige B., Spiegelhalder K., Nissen C., Voderholzer U., Lombardo C., Riemann D. (2011). Insomnia as a predictor of depression: A meta-analytic evaluation of longitudinal epidemiological studies. J. Affect. Disord..

[4] Bonnet M.H., Arand D.L. (2010). Hyperarousal and insomnia: State of the science. Sleep Med. Rev..

[5] Riemann D., Spiegelhalder K., Feige B., Voderholzer U., Berger M., Perlis M., Nissen C. (2010). The hyperarousal model of insomnia: A review of the concept and its evidence. Sleep Med. Rev..

[6] Kalmbach D.A., Anderson J.R., Drake C.L. (2018). The impact of stress on sleep: Pathogenic sleep reactivity as a vulnerability to insomnia and circadian disorders. J. Sleep Res..

[7] Harvey A.G. (2002). A cognitive model of insomnia. Behav. Res. Ther..

[8] Vgontzas A.N., Bixler E.O., Lin H.M., Prolo P., Mastorakos G., Vela-Bueno A., Kales A., Chrousos G.P. (2001). Chronic insomnia is associated with nyctohemeral activation of the hypothalamic-pituitary-adrenal axis: Clinical implications. J. Clin. Endocrinol. Metab..

[9] Winkelman J.W., Buxton O.M., Jensen J.E., Benson K.L., O’Connor S.P., Wang W., Renshaw P.F. (2008). Reduced brain GABA in primary insomnia: Preliminary data from 4T proton magnetic resonance spectroscopy (1H-MRS). Sleep.

[10] Kay D.B., Buysse D.J. (2017). Hyperarousal and Beyond: New Insights to the Pathophysiology of Insomnia Disorder through Functional Neuroimaging Studies. Brain Sci..

[11] Perlis M.L., Merica H., Smith M.T., Giles D.E. (2001). Beta EEG activity and insomnia. Sleep Med. Rev..

[12] Riedner B.A., Goldstein M.R., Plante D.T., Rumble M.E., Ferrarelli F., Tononi G., Benca R.M. (2016). Regional Patterns of Elevated Alpha and High-Frequency Electroencephalographic Activity during Nonrapid Eye Movement Sleep in Chronic Insomnia: A Pilot Study. Sleep.

[13] Feige B., Baglioni C., Spiegelhalder K., Hirscher V., Nissen C., Riemann D. (2013). The microstructure of sleep in primary insomnia: An overview and extension. Int. J. Psychophysiol..

[14] Merica H., Gaillard J.M. (1992). The EEG of the sleep onset period in insomnia: A discriminant analysis. Physiol. Behav..

[15] Baglioni C., Spiegelhalder K., Regen W., Feige B., Nissen C., Lombardo C., Violani C., Hennig J., Riemann D. (2014). Insomnia disorder is associated with increased amygdala reactivity to insomnia-related stimuli. Sleep.

[16] Pillai V., Roth T., Drake C.L. (2015). The nature of stable insomnia phenotypes. Sleep.

[17] Stepanski E., Zorick F., Roehrs T., Young D., Roth T. (1988). Daytime alertness in patients with chronic insomnia compared with asymptomatic control subjects. Sleep.

[18] Bonnet M.H., Arand D.L. (1995). 24-Hour metabolic rate in insomniacs and matched normal sleepers. Sleep.

[19] Roehrs T.A., Randall S., Harris E., Maan R., Roth T. (2011). MSLT in primary insomnia: Stability and relation to nocturnal sleep. Sleep.

[20] Colombo M.A., Ramautar J.R., Wei Y., Gomez-Herrero G., Stoffers D., Wassing R., Benjamins J.S., Tagliazucchi E., van der Werf Y.D., Cajochen C. (2016). Wake High-Density Electroencephalographic Spatiospectral Signatures of Insomnia. Sleep.

[21] Corsi-Cabrera M., Figueredo-Rodríguez P., del Río-Portilla Y., Sánchez-Romero J., Galán L., Bosch-Bayard J. (2012). Enhanced frontoparietal synchronized activation during the wake-sleep transition in patients with primary insomnia. Sleep.

[22] Corsi-Cabrera M., Rojas-Ramos O.A., del Río-Portilla Y. (2016). Waking EEG signs of non-restoring sleep in primary insomnia patients. Clin. Neurophysiol..

[23] Wu Y.M., Pietrone R., Cashmere J.D., Begley A., Miewald J.M., Germain A., Buysse D.J. (2013). EEG power during waking and NREM sleep in primary insomnia. J. Clin. Sleep Med..

[24] Sander C., Hensch T., Wittekind D.A., Bottger D., Hegerl U. (2015). Assessment of Wakefulness and Brain Arousal Regulation in Psychiatric Research. Neuropsychobiology.

[25] Hegerl U., Wilk K., Olbrich S., Schoenknecht P., Sander C. (2012). Hyperstable regulation of vigilance in patients with major depressive disorder. World J. Biol. Psychiatry.

[26] Morris S.E., Cuthbert B.N. (2012). Research Domain Criteria: Cognitive systems, neural circuits, and dimensions of behavior. Dialogues Clin. Neurosci..

[27] Jawinski P., Kirsten H., Sander C., Spada J., Ulke C., Huang J., Burkhardt R., Scholz M., Hensch T., Hegerl U. (2019). Human brain arousal in the resting state: A genome-wide association study. Mol. Psychiatry.

[28] Lane J.M., Jones S.E., Dashti H.S., Wood A.R., Aragam K.G., van Hees V.T., Strand L.B., Winsvold B.S., Wang H., Bowden J. (2019). Biological and clinical insights from genetics of insomnia symptoms. Nat. Genet..

[29] Vgontzas A.N., Fernandez-Mendoza J., Liao D., Bixler E.O. (2013). Insomnia with objective short sleep duration: The most biologically severe phenotype of the disorder. Sleep Med. Rev..

[30] (2013). World Medical Association Declaration of Helsinki: Ethical principles for medical research involving human subjects. JAMA.

[31] Iber C., Ancoli-Israel S., Chesson A., Quan S.F. (2007). The AASM Manual for the Scoring of Sleep and Associated Events: Rules, Terminology, and Technical Specifications.

[32] Bastien C.H., Vallieres A., Morin C.M. (2001). Validation of the Insomnia Severity Index as an outcome measure for insomnia research. Sleep Med..

[33] Buysse D.J., Reynolds C.F., Monk T.H., Berman S.R., Kupfer D.J. (1989). The Pittsburgh Sleep Quality Index: A new instrument for psychiatric practice and research. Psychiatry Res..

[34] Coren S., Mah K.B. (1993). Prediction of physiological arousability: A validation of the Arousal Predisposition Scale. Behav. Res. Ther..

[35] Nicassio P.M., Mendlowitz D.R., Fussell J.J., Petras L. (1985). The phenomenology of the pre-sleep state: The development of the pre-sleep arousal scale. Behav. Res. Ther..

[36] Johns M.W. (1991). A new method for measuring daytime sleepiness: The Epworth sleepiness scale. Sleep.

[37] Hoddes E., Zarcone V., Smythe H., Phillips R., Dement W.C. (1973). Quantification of sleepiness: A new approach. Psychophysiology.

[38] Waine J., Broomfield N.M., Banham S., Espie C.A. (2009). Metacognitive beliefs in primary insomnia: Developing and validating the Metacognitions Questionnaire—Insomnia (MCQ-I). J. Behav. Ther. Exp. Psychiatry.

[39] Schlotz W., Yim I.S., Zoccola P.M., Jansen L., Schulz P. (2011). The Perceived Stress Reactivity Scale: Measurement invariance, stability, and validity in three countries. Psychol Assess..

[40] Beck A.T., Ward C.H., Mendelson M., Mock J., Erbaugh J. (1961). An inventory for measuring depression. Arch. Gen. Psychiatry.

[41] Griefahn B., Künemund C., Bröde P., Mehnert P. (2001). Zur Validität der deutschen Übersetzung des Morningness-Eveningness-Questionnaires von Horne und Östberg. Somnologie.

[42] Huang J., Sander C., Jawinski P., Ulke C., Spada J., Hegerl U., Hensch T. (2015). Test-retest reliability of brain arousal regulation as assessed with VIGALL 2.0. Neuropsychiatr. Electrophysiol..

[43] Hegerl U., Hensch T. (2014). The vigilance regulation model of affective disorders and ADHD. Neurosci. Biobehav. Rev..

[44] Hirshkowitz M., Sharafkhaneh A., Kryger M., Roth T., Dement W.C. (2017). Evaluating sleepiness. Principles and Practice of Sleep Medicine.

[45] Horne J. (2010). Primary insomnia: A disorder of sleep, or primarily one of wakefulness?. Sleep Med. Rev..

[46] Dang-Vu T.T., Salimi A., Boucetta S., Wenzel K., O’Byrne J., Brandewinder M., Berthomier C., Gouin J.P. (2015). Sleep spindles predict stress-related increases in sleep disturbances. Front. Hum. Neurosci..

[47] Normand M.P., St-Hilaire P., Bastien C.H. (2016). Sleep Spindles Characteristics in Insomnia Sufferers and Their Relationship with Sleep Misperception. Neural. Plast..

[48] Forget D., Morin C.M., Bastien C.H. (2011). The role of the spontaneous and evoked k-complex in good-sleeper controls and in individuals with insomnia. Sleep.

[49] Wei Y., Colombo M.A., Ramautar J.R., Blanken T.F., van der Werf Y.D., Spiegelhalder K., Feige B., Riemann D., Van Someren E.J.W. (2017). Sleep Stage Transition Dynamics Reveal Specific Stage 2 Vulnerability in Insomnia. Sleep.

[50] Christensen J.A.E., Wassing R., Wei Y., Ramautar J.R., Lakbila-Kamal O., Jennum P.J., Van Someren E.J.W. (2019). Data-Driven Analysis of EEG Reveals Concomitant Superficial Sleep During Deep Sleep in Insomnia Disorder. Front. Neurosci..

[51] Bastien C.H., St-Jean G., Turcotte I., Morin C.M., Lavallee M., Carrier J. (2009). Sleep spindles in chronic psychophysiological insomnia. J. Psychosom. Res..

[52] Bastien C.H., St-Jean G., Turcotte I., Morin C.M., Lavallée M., Carrier J., Forget D. (2009). Spontaneous K-complexes in chronic psychophysiological insomnia. J. Psychosom. Res..

[53] Riemann D., Klein T., Rodenbeck A., Feige B., Horny A., Hummel R., Weske G., Al-Shajlawi A., Voderholzer U. (2002). Nocturnal cortisol and melatonin secretion in primary insomnia. Psychiatry Res..

[54] Drake C.L., Pillai V., Roth T. (2014). Stress and sleep reactivity: A prospective investigation of the stress-diathesis model of insomnia. Sleep.

[55] Palagini L., Mauri M., Dell'Osso L., Riemann D., Drake C.L. (2016). Trait- and pre-sleep-state-dependent arousal in insomnia disorders: What role may sleep reactivity and sleep-related metacognitions play? A pilot study. Sleep Med..

[56] Kalmbach D.A., Cuamatzi-Castelan A.S., Tonnu C.V., Tran K.M., Anderson J.R., Roth T., Drake C.L. (2018). Hyperarousal and sleep reactivity in insomnia: Current insights. Nat. Sci. Sleep.

[57] Blanken T.F., Benjamins J.S., Borsboom D., Vermunt J.K., Paquola C., Ramautar J., Dekker K., Stoffers D., Wassing R., Wei Y. (2019). Insomnia disorder subtypes derived from life history and traits of affect and personality. Lancet Psychiatry.

